# Bottom-Up Regulation of Capelin, a Keystone Forage Species

**DOI:** 10.1371/journal.pone.0087589

**Published:** 2014-02-04

**Authors:** Alejandro D. Buren, Mariano Koen-Alonso, Pierre Pepin, Fran Mowbray, Brian Nakashima, Garry Stenson, Neil Ollerhead, William A. Montevecchi

**Affiliations:** 1 Cognitive and Behavioural Ecology Programme, Memorial University, St. John’s, Newfoundland and Labrador, Canada; 2 Northwest Atlantic Fisheries Centre, Fisheries and Oceans Canada, St. John’s, Newfoundland and Labrador, Canada; Aristotle University of Thessaloniki, Greece

## Abstract

The Northwest Atlantic marine ecosystem off Newfoundland and Labrador, Canada, has been commercially exploited for centuries. Although periodic declines in various important commercial fish stocks have been observed in this ecosystem, the most drastic changes took place in the early 1990s when the ecosystem structure changed abruptly and has not returned to its previous configuration. In the Northwest Atlantic, food web dynamics are determined largely by capelin (*Mallotus villosus*), the focal forage species which links primary and secondary producers with the higher trophic levels. Notwithstanding the importance of capelin, the factors that influence its population dynamics have remained elusive. We found that a regime shift and ocean climate, acting via food availability, have discernible impacts on the regulation of this population. Capelin biomass and timing of spawning were well explained by a regime shift and seasonal sea ice dynamics, a key determinant of the pelagic spring bloom. Our findings are important for the development of ecosystem approaches to fisheries management and raise questions on the potential impacts of climate change on the structure and productivity of this marine ecosystem.

## Introduction

The Northwest Atlantic is a highly productive low-Arctic ecosystem that has supported commercial fishing activities for more than half a millennium. The structure of the food web is best described as a wasp-waist pattern, in which a crucial intermediate trophic level is dominated by a single species [1]. The dynamic properties of wasp-waist food webs are critically determined by the species at the waist [2]. Capelin (*Mallotus villosus*) fulfils this role in the Northwest Atlantic, acting as a link between zooplankton and large vertebrates [3]. Consequently, elucidating the mechanisms that regulate capelin populations is important to understanding the dynamics of the system. Trophic control of ecosystems is often described in terms of bottom-up (resource-driven) or top-down (consumer-driven) regulation, though these are just extremes on a continuum; the most parsimonious description is that control is spatially and temporally variable [4].

The marine community off the Newfoundland and Labrador Shelf underwent a series of radical changes during the early 1990s; abundance of Atlantic cod (*Gadus morhua*), the dominant groundfish and major predator of the system collapsed [5]–[7] while much of the demersal fish community suffered an overall decline [5], [8]–[11], and shellfish biomass increased [12], [13]. These changes were accompanied by the ongoing rebuilding of the harp seal (*Pagophilus groenlandicus*) population [14], [15], and shifts in the diets, phenologies and population trends of seabirds [16], [17]. The capelin stock suffered a major biomass decline in 1991, from which it has not yet recovered [18]; spawning became protracted and was delayed up to four weeks [18], [19], while size and age at maturity, and somatic condition declined [20], [21].

Extensive, and sudden, changes in marine ecosystems such as those observed during the early 1990s on the Newfoundland-Labrador Shelf are usually linked to regime shifts [22]–[25]. These are defined as rapid, pervasive, and persistent changes in system structure forced by environmental perturbation that alter key energy pathways [26]. A climate-induced regime shift occurred in the North Atlantic during the 1920s and 1930s, when significant changes in several marine ecosystems of the northern North Atlantic were linked to general ocean warming [27]. During 1991, a pulse of fresh water flowing from the Arctic [28] created unusual climatic conditions in the area; the water temperature was the coldest in 50 or more years, reaching a centennially significant nadir [29]. The synchrony of this perturbation in the climatic record with the restructuring of the community raises the possibility that they are related.

In this study we focus on the regulating mechanisms of the keystone forage species in the system, punctuated by the extreme events of the early 1990s. Despite its role as the main channel of energy flow between basal trophic levels and top predators, little is known about the factors that regulate the capelin population. Given the sudden nature of the changes in capelin during the 1990s, the small magnitude of the capelin fishery [18], and the expected predation release from a declining groundfish assemblage, changes in capelin were likely driven by bottom-up effects, though the mechanisms involved are unknown. Year-class strength is, however, regulated by meteorological and hydrographical variables [30].

Copepods, mainly *Calanus finmarchicus*, are the most important prey species for capelin in the Northwest Atlantic [31], [32]. At high latitudes, calanoid copepods accumulate large lipid reserves [33], which likely fuel the growth of predators (e.g. capelin). Copepod production is regulated by physical and biological variables, though their relative strengths are heterogeneous in space and time [34], [35]. *Calanus finmarchicus* production off Newfoundland is affected by temperature and cannibalism by adult females [34], which increases when phytoplankton abundance is low [36]. Thus, food availability in spring is likely a limiting factor for *C. finmarchicus* production on the Newfoundland–Labrador Shelf.

The spring bloom on the shelf follows the retreat of seasonal sea ice. Arctic sea ice flows southward onto the Newfoundland-Labrador Shelf during winter. During early spring accelerated melting causes the ice edge to retreat northward and the freshwater runoff is advected by the Labrador Current onto the Grand Banks, causing rapid stratification of the water column and promoting phytoplankton growth in the shallow mixed layer [37].

The objectives of this study are to assess the occurrence of a regime shift during the early 1990s on the Newfoundland-Labrador Shelf marine ecosystem, and examine the effects of sea ice on capelin population biomass and timing of spawning to examine the hypothesis that capelin is environmentally regulated via food availability.

## Materials and Methods

### Data Series

#### Climate

**Figure 1:**
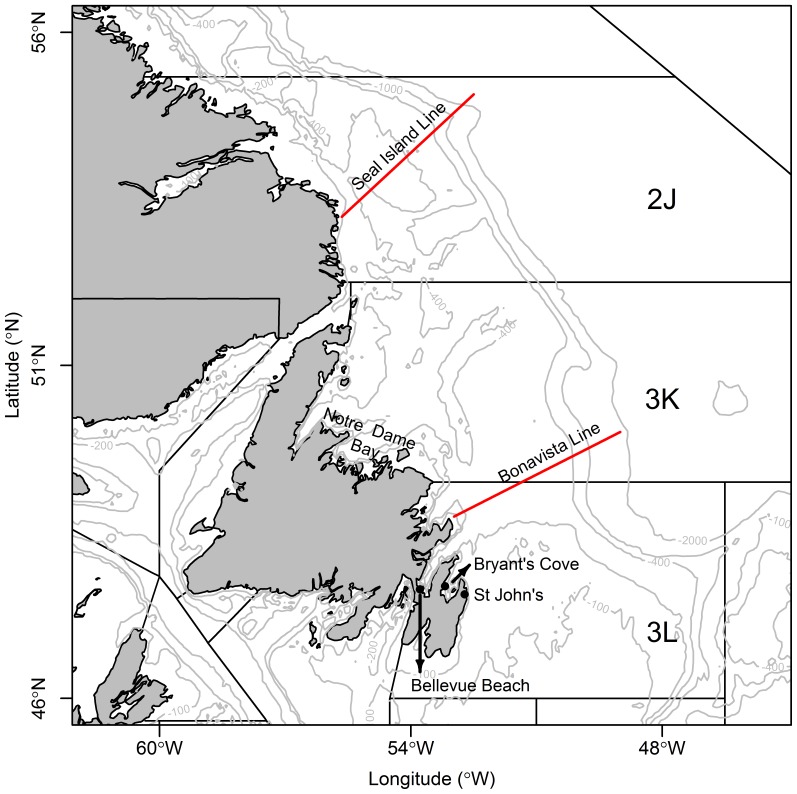
Study area. Capelin stock area, NAFO Divisions 2J3KL. The locations of St John’s, Bryant’s Cove, Bellevue Beach, Notre Dame Bay, and the *Atlantic Zone Monitoring Program (AZMP)* lines Seal Island and Bonavista are indicated.

To assess environmental patterns linked to the climatic perturbation of the 1990s we examined 5-year running means of air temperature anomalies and their cumulative sums - the methodology used previously to describe the climate forcing associated with the North Atlantic regime shift of the 1920s and 1930s [27]. We examined air temperatures anomalies (from the long-term mean; since 1874) recorded at the monitoring station in St. John’s, Newfoundland (Figure 1) (http://www.meds-sdmm.dfo-mpo.gc.ca/isdm-gdsi/azmp-pmza/climat/airTemp-eng.asp?stn=STJOHNS). We also examined the Atlantic Multidecadal Oscillation (AMO) anomalies and their cumulative sums (from the long-term mean; since 1856) (121-month smoothed estimates as provided by NOAA http://www.cdc.noaa.gov/Timeseries/AMO). The AMO is an ongoing series of oscillatory changes in basin-wide North Atlantic sea-surface temperature with a period of 65–70 years [38] **.**

#### Sea ice

Weekly data on sea ice concentration of various thicknesses for 1969–2010 were obtained from the Canadian Ice Service (CIS) (http://www.ice-glaces.ec.gc.ca/). Two parameters were derived from each map: southernmost position of the ice edge (10% total ice concentration) and ice-covered area south of 55°N. Occasionally, ice sheets which are clearly not part of the ice pack that drift southward from the Arctic are retained in Notre Dame Bay (Figure 1), and were thus not considered in the derivation of the sea ice parameters. The maximum value of ice area south of 55°N was then extracted for each year (A_ice_), and start time of ice retreat (t_ice_) was considered to be the day when the ice edge reached the southern-most latitude in a given ice season (November–July).

#### Capelin

We used a capelin stock biomass index derived from Fisheries and Oceans Canada’s (DFO) spring acoustic survey [methodological details in 39]. Given its typical pelagic behaviour, standard random-stratified bottom trawl surveys are not the best method to estimate capelin abundance; although the introduction of the Campelen gear in DFO’s research surveys in the region in 1995 [40], [41] significantly improved the survey performance for small fishes, including capelin. Notwithstanding these improvements, the most reliable method for estimating capelin abundance is acoustic integration supported by directed trawling [42].In this context, there are no reliable estimates of spring spawning biomass for the entire stock that expands before and after the 1991 biomass decline, but an index covering the core of the historical spring distribution area is available from the DFO’s spring acoustic survey [18]. Since this survey has only partial coverage of the entire stock area, the estimates it provides are considered to be minimum biomass estimates. Nonetheless, the estimates from this survey show a high degree of internal consistency, and thus the relative biomass index for the capelin stock in the region can be considered reliable [43]. Directed trawling is used to assess the age structure of the stock being measured acoustically. Due to gear’s size dependent catchability, age 1 capelin has been poorly represented and, age 2, followed by age 3 fish accounted for the majority of fish caught in most years [43]. Historically, the spawning populations were composed of mainly three and four year old fish. However, since the early 1990s spawning populations have consisted predominantly of two and three year old fish, with the percent maturing of two year-old reaching almost as high as 80% [43], [44]. Capelin biomass estimates from DFO’s spring acoustic survey are available for the years 1982, 1985–1992, 1996, 1999–2005 and 2007–2010. Monte Carlo simulations were implemented to estimate 95% confidence intervals [methodological details in 43]. Raw acoustic data were not available for years prior to 1988; hence confidence limits could not be calculated.

To examine the difference in the timing of spawning, we used a long term data set from two beach sites in Newfoundland [methodological details in 45]. The Annual date of peak spawning has been recorded systematically at Bryant’s Cove, 1978–2010 and Bellevue Beach, 1990–2010 (Figure 1 ) [18].

#### Prey of capelin

Abundance estimates of adult Calanus finmarchicus (6^th^ copepodite stage) were based on collections from DFO Atlantic Zone Monitoring Programme (AZMP) (http://www.meds-sdmm.dfo-mpo.gc.ca/isdm-gdsi/azmp-pmza/index-eng.html) along two oceanographic sections on the Newfoundland-Labrador Shelf (Figure 1). Data are available from 1999–2010 only, and thus effects of C. finmarchicus availability on capelin biomass and/or timing of spawning could not be assessed directly. Complete details of field and laboratory protocols are available in Mitchell et al [46]. We focussed analyses on the abundance of the adult stages (6^th^ copepodite stage, CVI) of C. finmarchicus because capelin feed predominantly on these larger copepods [32] which are most abundant, in terms of numbers and overall proportion of the population, in the late autumn and early spring (i.e. prior to the surveys from which capelin abundance are derived). Annual estimates (1999–2010) of water column inventories of adult Calanus finmarchicus from the Shelf stations (depth <400 m) along the Bonavista and Seal Island lines were based on general linear models of the form ln(Density)∼year+station+season for each oceanographic section, where Density is in units of m^−2^, based on type III sums of squares estimates of overall year effect [47].

### Analyses

Given the abrupt changes observed in the Newfoundland and Labrador Shelf ecosystem in the early 1990s (presented in Introduction), we included in all analyses a categorical variable “period” to all our analytical models to discriminate the conditions in the last 2 decades (1991–2010) from those before 1991. The timing of peak spawning at Bryant’s Cove and Bellevue Beach and capelin biomass index were described as functions of “period” and ice parameters. In all cases, ice parameters used were the annual maximum extent of ice *A_ice_* and start time of ice retreat *t_ice_*, although we only show the ice-capelin relationships with the parameters that yielded the best fit to the data.

#### Capelin biomass

To describe the variations in capelin biomass as a function of the timing of the onset of the spring bloom (triggered by sea ice retreat), we fitted a linear model and two different formulations of a dome-shaped model. We discarded the linear model given that it did not impose an upper bound on the estimated capelin biomass, i.e. as the spring bloom occurred later in the year capelin biomass grew unbounded to extreme levels. Logically, there must be an optimum timing of the spring bloom that yields a maximum of capelin biomass, with either an early or late spring bloom negatively affecting biomass. The difference between the dome-shaped models we used resides in the descending limbs of the functions (i.e. during summer and autumn); if the start time of ice retreat occurs past the optimum capelin biomass in one of the functions declines to some extent, while in the second it eventually reaches zero. Given that the explanatory variable we are using is a proxy for the timing of the spring bloom, we only have information on the ascending limbs of the functions and thus it is not possible to inform the model with respect to the effect of the start time of ice retreat on capelin biomass if the retreat occurs either in summer or fall. The models had an almost identical fit (not shown here) and thus we decided to present the model where capelin biomass reaches zero, based on the premise that if the bloom does not occur by fall, there will be no food supply available for capelin and thus the biomass will drop to zero. Therefore, the dome-shaped model we used to capture the patterns of variation in capelin biomass had the form
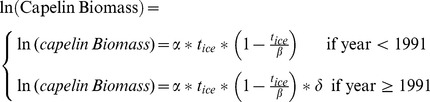
where *α* and *β* are the parameters that determine the shape of the dome and *δ* is a scale parameter that represents the effect of “period”. The optimum start time of ice retreat (i.e. timing that results in maximum capelin biomass) is 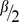. We assumed a normal multiplicative observation error (on the logarithmic scale), and fit the model by minimizing the negative log-likelihood function using the “nlminb” function in R [48]. The maximum likelihood estimates of the parameters were used to produce a hindcast of capelin biomass predicted from the values of *t_ice_* since 1972. These predictions were compared with estimates of capelin biomass on January 1^st^ 1972–1980 produced by sequential capelin abundance models [SCAM; 49]. These models are similar to VPA and cohort analysis, and were used in the past to provide management advice [49]. We also forecast the capelin biomass and compared the prediction to the acoustic estimate for 2011.

#### Capelin spawning

To describe the relationship between spawning timing and sea ice, we fitted a general linear model of the form peakdate∼ A_ice_+ beach+period, where beach is either Bryant’s Cove or Bellevue Beach. We did not include the peak spawning date in 1990 at Bellevue Beach, as this was the only datum available for the period pre-1991. To assess the significance of A_ice_ we constructed an empirical frequency distribution by bootstrapping the residuals within each period and beach (n_boot_ = 500000). Maximum likelihood parameter estimates were used to produce forecast spawning dates in 2011, and these were compared to field observations.

#### Prey availability

If capelin is regulated by bottom-up processes from the base of the food web, we would expect to find a relationship between the timing of retreat of sea ice (as a proxy of the timing of onset of the spring bloom) and the abundance of capelin’s main prey, C. finmarchicus. To explore this relationship, we fitted a general linear model of the form ln(abundance Calfin)∼ t_ice_+line, where line refers to the AZMP lines where abundance was estimated: Seal Island and Bonavista (Figure 1). To assess the significance of t_ice_ we constructed an empirical frequency distribution by bootstrapping the residuals within each line (n_boot_ = 500000).

## Results

### Climate

**Figure 2:**
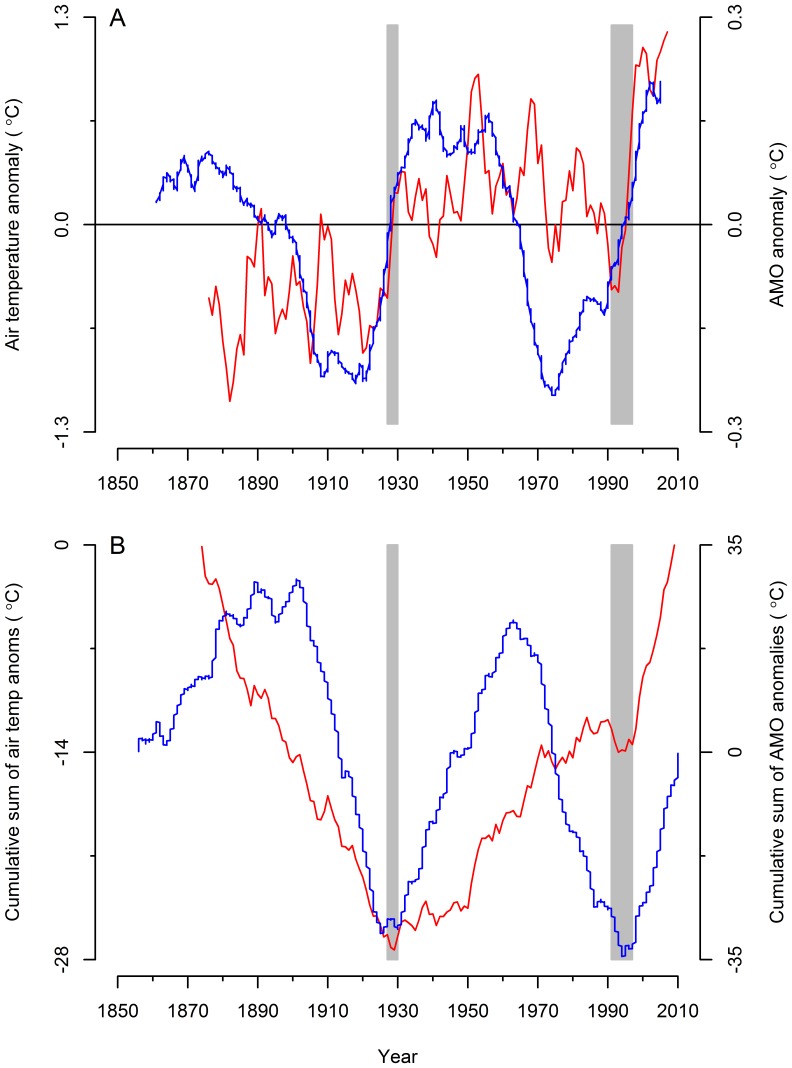
Climatic patterns. (A) 5-year running mean of air temperature anomalies in St John’s (red line) and 121-month smoothed Atlantic Multidecadal Oscillation (AMO) anomalies (from long term means), (B) Cumulative sums of air temperature anomalies in St John’s (red line) and Atlantic Multidecadal Oscillation anomalies (blue line). Shaded portions represent periods when regime shifts occurred.

Air temperature at St John’s (Figure 2) was relatively cool during the latter part of the 19^th^ century and the beginning of the 20^th^, compared to temperatures during the mid-20^th^ century and especially compared to the latter part of the 20^th^ and beginning of the 21^st^ centuries. The cumulative sum of air temperature anomalies abruptly changed sign in 1929 (Figure 2). During the early 1990s, air temperature was substantially colder than average, though by 1996 it increased rapidly and has since remained at above average values.

The AMO shows a periodicity of ∼65 years (Figure 2). Notably, the minimum points of the cumulative sum of the AMO cycle (when the system switches from a cool to a warm phase) coincided with the transitional periods in the air temperature time series (Figure 2).

### Sea Ice

**Figure 3:**
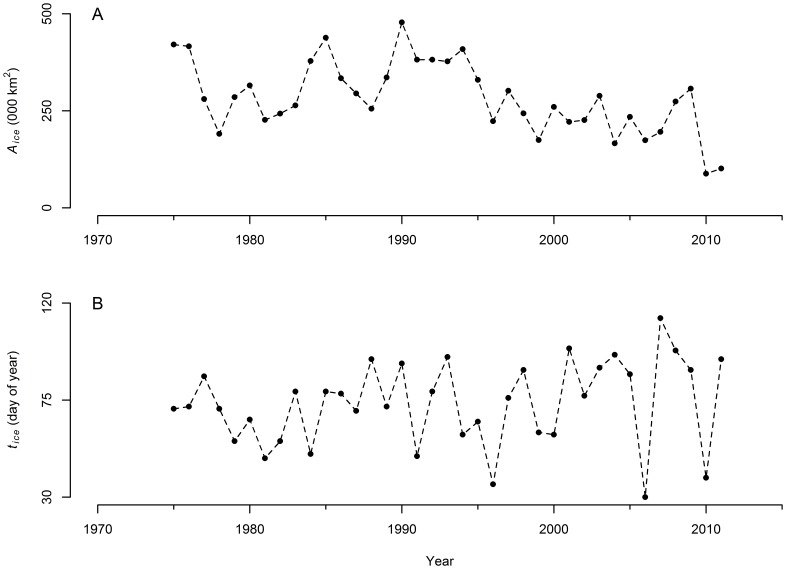
Sea ice properties. (A) Maximum annual extent of sea ice *A_ice_*, (B) start time of ice retreat *t_ice._*

Maximum annual extent of sea ice is highly variable, ranging from ∼150,000 to ∼475,000 km^2^ (Figure 3A), with the exception of 2010 and 2011, when *A_ice_* was at its minimum at 88,000 and 101,000 km^2^ respectively. Variability in *A_ice_* has however been declining since the mid-1990s (Figure 3A).

The timing of the retreat of sea ice varies between late January and mid-April (Figure 3B). In only a few years (1981, 1984, 1991, 1996, 2006, 2010) the retreat of sea ice occurred before February 19 (day 50 of the year). The southernmost extent of ice occurred in 1991, when a narrow ice tongue extended as far south as Boston, USA (42° 28′ N), and there was ice present off St John’s until mid-May. It is noteworthy that both the earliest (January 30) and latest (April 23) ice retreat dates occurred in 2006 and 2007 respectively (Figure 3B).

### Capelin Biomass

**Figure 4:**
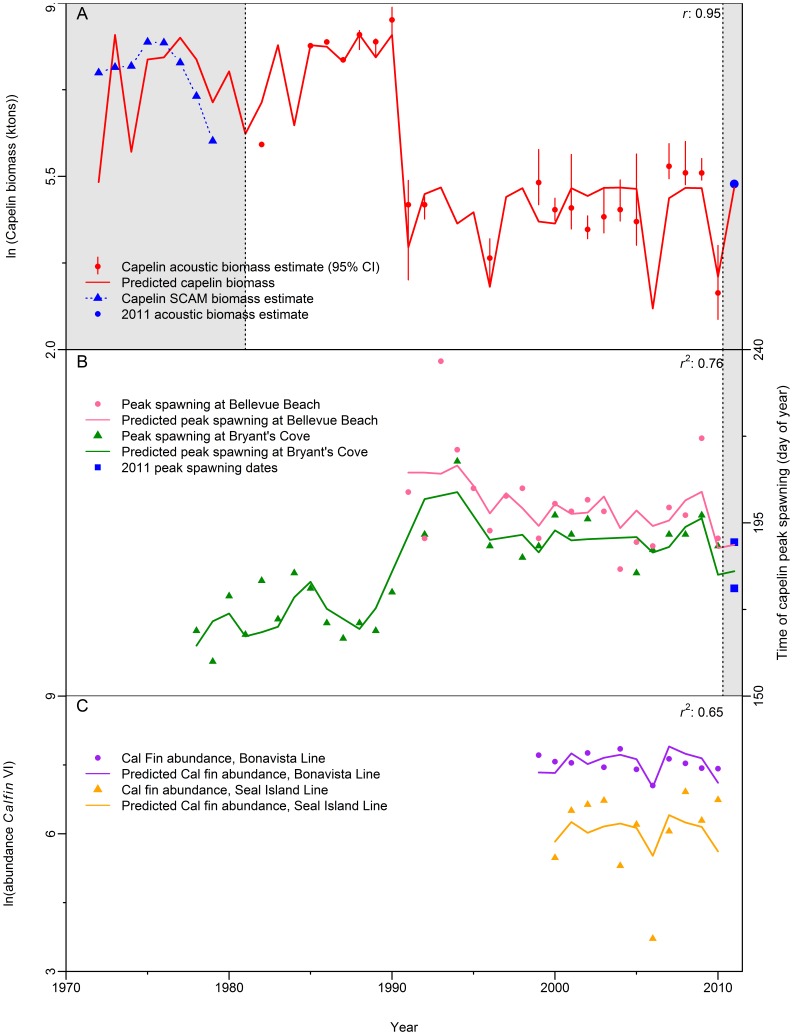
Relationship between biological variables, ice characteristics and “period”. Capelin and *Calanus finmarchicus* characteristics explained by ice properties and “period”. (A) Capelin biomass. (B) Timing of capelin peak spawning date at Bryant’s Cove and Bellevue Beach. (C) Abundance of adult *C. finmarchicus* (Calfin VI) on the Newfoundland-Labrador Shelf. Shaded portions indicate models forecasts and hindcast contrasted to independent data.

Acoustic estimates of capelin biomass (Figure 4A) range from 466,000 tonnes (1982) to 5,783,000 tonnes (1990) in the pre-1991 time series. In 1991, the acoustic estimate dropped to 138,000 tonnes (∼10% of historical values) and oscillated around 100,000 tonnes until 2007 when it increased slightly to 300,000 tonnes and remained at a similar level until 2010 when it declined to the lowest estimate in the time series (23,000 tonnes, ∼1% of historical values) (Figure 4A).

**Figure 5:**
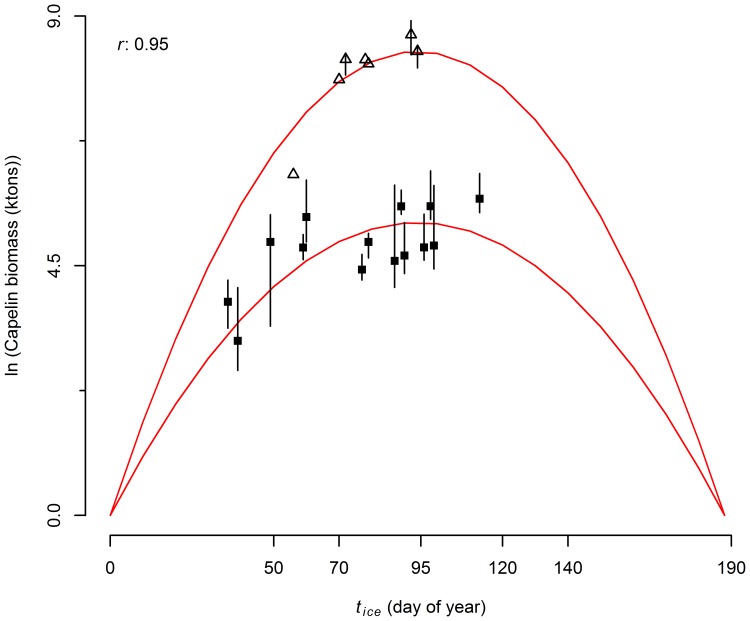
Fit of the dome-shaped capelin biomass model. Open triangles denote capelin acoustic biomass estimates prior to 1991 and filled squares during the post 1990 period. Bars denote 95% confidence intervals. Note that y-axis is in logarithmic scale.

We found that variations in capelin stock biomass estimates from spring acoustic surveys were well explained by the dome-shaped model (Figure 4A, *r* = 0.95, *n* = 21). Maximum capelin biomass occurs when the sea ice retreats northward in early April (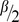 = 93.95 = April 4); low biomasses are expected if ice retreats earlier than February 19 (Figure 5). Capelin biomass hindcasts for 1972–1980 agreed well with earlier estimates obtained from SPA analyses (Figure 4A). Considering that these earlier estimates were produced with a different methodology and for the beginning of the year [49] (unlike the acoustic surveys modeled by our analysis, which are carried out during May–June), the fact that both sets of model predictions show similar trends is important and reassuring. Moreover, the model forecast for 2011 (196,000 tonnes) and the survey acoustic estimate for that year (210,000 tonnes, not used to fit the model) also showed excellent concurrence.

### Capelin Spawning

Capelin’s peak spawning date at Bryant’s Cove during the first years of the time series (until 1990) ranged from June 7 (1979) to June 30 (1984) (Figure 4B). In the later period, peak spawning date was delayed by about a month, ranging from July 1 (2005) to July 30 (1994). The peak spawning date at Bellevue Beach reflected that at Bryant’s Cove, occurring on July 2 (1990) and ranging between July 1 (1994) and August 5 (2009). Peak spawning occurred particularly late in 1993, August 8 (Figure 4B).

A large proportion of the variability in the date of peak spawning at Bellevue Beach and Bryant’s Cove was explained by the general linear model (Figure 4B, p*_Aice_* = 0.000008, *r^2^* = 0.76, *n* = 48). Furthermore, there was strong agreement between the predicted and observed dates of peak spawning for 2011, a 1- and 4-day difference for Bellevue Beach and Bryant’s Cove, respectively (Figure 4B).

### Prey Availability

We found a positive and significant relationship between the density of adult *C. finmarcihus* and the timing of retreat of sea ice (Figure 4C, p*_tice_* = 0.01, *r^2^* = 0.65, *n* = 23).

## Discussion

Capelin exhibited an abrupt state change in the early 1990s that is consistent with a regime shift (see below). We further show that physical factors in the form of seasonal sea ice dynamics regulate the timing of spawning and the population biomass trajectory of capelin in the Northwest Atlantic.

In our analysis of a long-term time series (>20 years) of capelin population biomass and time of peak spawning in waters off Newfoundland, we explained the time of peak spawning as a linear function of the maximum annual extent of sea ice (*A_ice_*) and capelin biomass as a dome-shaped function of the start time of ice retreat (*t_ice_*). In both cases we included a break at the year 1991 to account for the extensive changes that occurred in the marine community on the Newfoundland-Labrador Shelf. It is noteworthy that seasonal sea ice dynamics (although different characteristics) drive both biomass and timing of spawning, and that in both cases the year-to-year relationships are not changed after the tipping point in 1991; rather they are merely shifted down in the case of biomass and toward later dates in the case of timing of peak spawning. This suggests that, despite the drastic changes in capelin in particular and the system in general, the mechanisms that modulate timing of peak spawning and stock biomass have remained unchanged.

Carscadden et al. [45], [50] proposed that capelin spawning time could be explained by a combination of fish length and an integration of the temperature in the upper 20 m of the water column during February–June (TEMPSUM). These authors hypothesized that temperature modulates spawning time via its effect on zooplankton abundance and on the rates of gonadal development. Our explanation of the impact of the environment on spawning time differs from Carscadden et al.’s [45], [50] hypothesis (based on data through 1994). We assessed the relationship between timing of spawning at Bryant’s Cove and TEMPSUM including data from 1978 to 2009 and found that the relationship breaks down (*r* = 0.06, data not shown), rejecting the hypothesis that temperature regulates spawning timing via accelerated gonad development in warmer years. Our analyses indicate that timing of spawning can be described by a combination of the maximum annual extent of sea ice (*A_ice_*) and a categorical variable “period”, which effectively separates pre-1991 data from the last 20 years of the time series. We found that *A_ice_* and TEMPSUM are negatively correlated (*r* = −0.75, data not shown), i.e. the annual maximum extent of sea ice is larger in colder years, which explains the transient positive relationship between temperature and timing of spawning Carscadden et al found [45]. They reported lengths in the 160–180 mm range prior to 1991 and 140–160 mm range thereafter (Figure 5 in Carscadden et al. [45]). Subsequent analysis indicates that lengths have remained in the 140–160 mm range [18] suggesting that a shift occurred in 1991, rather than a continuous relationship between spawning timing and capelin length. Though the mechanisms that regulate the timing of spawning are yet not clear, it is likely that fall (which determine the amount of energy reserves maturing capelin accumulate) and spring feeding conditions (which are related to the way sea ice impacts the phytoplankton spring bloom) interact to influence spawning time.

Our biomass model captured biomass values very well and, most importantly, the temporal trends (Figure 4A). The only year when the model behaved somewhat poorly was 1999. A number of unusual biological events occurred in 1999 [51]–[53], including the occurrence of the Pacific diatom *Neodenticula seminae* on the Labrador Shelf for the first time in 800,000 years [54]. The reliability of our capelin biomass model was explored by a) generating a hindcast and contrasting these predictions with existing estimates of a sequential capelin abundance model and, b) forecasting the 2011 biomass (196,000 tonnes) which could be compared to the acoustic estimate (210,000 tonnes). The reasonable agreement between the data and hindcast and good agreement with the forecast projections support our identification of plausible mechanisms that regulate capelin biology. That is 1) a regime shift in the early 1990s, and 2) seasonal sea ice dynamics as a regulator of primary production on the Newfoundland and Labrador Shelf (its impact percolating through the food web via *Calanus finmarchicus* to capelin).

### The Regime Shift

In addition to continuous state changes, ecosystems may undergo punctuated, drastic shifts when environmental conditions cross a threshold tipping point [55]. In this paper we used a variable “period” to explain the biological changes that occurred in the Northwest Atlantic. Here we link this sudden change in biological variables to environmental forcers, and, propose that a regime shift occurred on the Newfoundland-Labrador Shelf during the early 1990s.

Two clear breaks occurred in the data series of meteorological patterns we analyzed (late 1920s and early 1990s). In addition, the largest annual decline in the North Atlantic Oscillation (NAO) was recorded in 1996 [56], synchronously with the second break. This suggests the occurrence of two regime shifts, the first in the late 1920s which is consistent with Drinkwater’s [27] description, and the second during the early to mid-1990s, characterized by a brief period (1991–1995) of transient climatic forcing. The concomitant restructuring of the system described above (collapse of groundfish stocks, increase in shellfish, extensive changes in capelin biology, rebuilding of harp seal population, changes in seabirds’ biology), and the persistence of this new state are consistent with the definition of regime shifts.

We interpret the 1990s regime shift as the result of synergistic climatic and anthropogenic forcers. The system has been intensively exploited for centuries, targeting top predators (*e.g.* Atlantic cod) significantly reducing their stock sizes [57]. Apex predators link multiple sub-systems in complex food webs conveying system stability [58], [59]. Thus, these large removals likely eroded the system’s resilience, possibly paving the way for major changes when exposed to a punctuated extreme event [55]. Climate change altered Arctic circulation patterns leading to enhanced low-salinity export into the Northwest Atlantic [28]. A major pulse of surface water flowed from the Arctic through the Canadian archipelago reaching the Northwest Atlantic in 1991, causing a regime shift in the Georges Bank ecosystem [28], [60]. This rare event, acting on a system already under stress, likely affected the state of the Newfoundland-Labrador Shelf ecosystem, moving it to a different configuration [8], [10], [11]. The reversal in the trend of atmospheric variables by the mid-1990s (Figure 2) potentially affected the currently dominant ecosystem configuration.

### The Capelin Regulating Mechanism

We hypothesize that the mechanistic linkage between sea ice and the modulation of capelin is a match/mismatch phenomenon between the timing of the onset of the spring bloom, triggered by the retreat of sea ice [37], and the abundance of emergent *Calanus finmarchicus* (capelin’s main prey) from diapause, with its effects percolating to capelin via nutritional stress.

**Figure 6:**
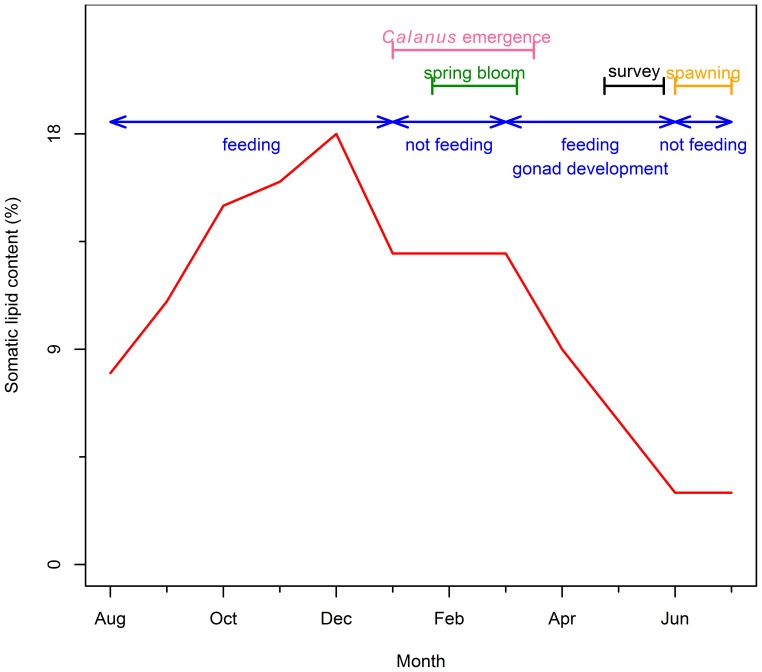
Schema of chronology of events relevant to the phenology of capelin. Somatic lipid contents of adult capelin (red line) [redrawn from Table 1 in 61], periods of capelin feeding and gonadal development (blue line) [62], timing of the spring bloom (green line) [37], timing of *Calanus finmarchicus* emergence from dormancy (pink line) [71], timing of capelin spawning (orange) and timing of capelin acoustic survey (black line).

Capelin feed in the late summer and autumn, building up their somatic lipid reserves which reach their maximum by the end of the year [61, Figure 6]. During winter (January-March) capelin do not feed and concentrate in large, inactive schools in cold water [62]. Gonad development and maturation begins in early April, when capelin concentrations move toward warming surface waters to feed. Somatic lipid reserves are moved to the gonads, decreasing somatic condition through the spring, and reaching a minimum prior to spawning (Figure 6), making this a key period in their phenology. The timing of the DFO acoustic capelin surveys (May–June, Figure 6) is ideal for capturing the realisation of processes that occur during spring. We hypothesize that if capelin do not find an abundant food source during this critical stage, it would lead to augmented natural mortality either via starvation or enhanced susceptibility to predation and reduced competitive abilities. An alternative explanation would be that as a result of low *Calanus* abundance (during years when the ice retreats early) capelin may redistribute. Distribution and migratory patterns of Barents Sea capelin depend on stock size, as a large stock leads to food depletion and consequent relocation to meet higher food demands [63], [64]. As a result of relocating, capelin may either not spawn at all or spawn elsewhere and the resulting progeny would be lost to the stock, along with the progenitors due to the high level of post spawning mortality [65]–[68].

*Calanus finmarchicus* feed intensively on phytoplankton during summer accumulating large lipid stores. Later in the season they sink into deep water and undergo diapause, a phase of arrested development and reduced metabolism [69]. In spring, the dormant stages re-emerge and migrate to surface waters taking advantage of the spring phytoplankton bloom to support high reproductive rates [70]. Emergence on the Newfoundland-Labrador Shelf is highly variable and begins before the spring chlorophyll peak [71], from late February to late April [36]. Adult and offspring *Calanus* survival will be enhanced if this period coincides with the peak spring bloom; adults will have an improved body condition and offspring will be subjected to lowered rates of cannibalism. If, on the other hand, the onset of the bloom occurs too early in the season, *Calanus* will likely emerge too late to fully utilize the high chlorophyll concentrations during the bloom, resulting in low *Calanus* biomass. The timing of the spring bloom on the Newfoundland-Labrador Shelf is determined by the timing of retreat of seasonal sea ice from the area [37]. If match/mismatch were the regulating mechanism, we would expect the positive relationship between timing of retreat of sea ice and *C. finmarchicus* abundance in the spring which was observed (Figure 4C).

These results indicate that the energy flow in the Newfoundland Shelf ecosystem seems to be controlled by bottom-up processes. This finding is consistent with the form of regulation of many forage fish, which show strong and rapid population responses to environmental variability [25], [72]–[75]. Our bottom-up regulation hypothesis contrasts with Frank et al.’s [4] proposal that the control is exerted from the top-down. The foundation for their assertion is a negative correlation between the abundance of benthic and forage fish species from 1970–1994, as measured by scientific bottom trawl surveys (their Figure 3A). They describe the abundance of forage fish as oscillating until the mid-1980s and increasing thereafter. Because capelin is the most abundant forage fish in the system, their “forage fish” signal must be driven primarily by the abundance of capelin, so this figure contradicts the accepted view of capelin’s history (Figure 4A) [18]. The reason for this discrepancy lies in the methodology used to estimate capelin abundance on the Newfoundland-Labrador Shelf. Frank et al. [4] estimated forage fish abundance from bottom trawl survey data, but the Campelen trawl, which improved the ability of Newfoundland and Labrador DFO bottom trawl surveys to estimate small fishes, was only introduced in 1995. Prior surveys in the region, which were the ones used by Frank et al. [4], used the Engels trawl, which has a known poor performance for catching small fishes, making them an unreliable source for estimating capelin abundance.

Capelin is a key prey for many predators in the system, such as cod [76]–[78], harp seals [79], Greenland halibut (*Reinhardtius hippoglossoides*) [80], whales (Lawson and Stenson, Fisheries and Oceans, unpublished data) and seabirds [81]–[83]. Although these predators consume large amounts of capelin annually [79], [84], [85], the trends in capelin abundance and consumption are not consistent with the theory of top-down control. Further, predator consumption does not change instantly and therefore cannot account for the sudden change in capelin abundance observed during the 1990s. Based upon our model, the effects of bottom-up environmental forcers account for over 90% of the variation in the time series of stock biomass indicating that the capelin population is not regulated by top-down mechanisms.

The Newfoundland-Labrador Shelf ecosystem is similar in some respects to the Norwegian and Barents Seas and the Iceland basin that are influenced by Atlantic and Arctic currents, and have relatively simple structures, with one main group of zooplankton *Calanus* spp., pelagic forage fish (capelin, herring, blue whiting (*Micromesistius poutassou*)), and demersal fish (most prominently Atlantic cod), marine mammals, and seabirds at the top of the food web. As in the Newfoundland-Labrador Shelf ecosystem, physical forcers play substantial roles in regulating these northern marine ecosystems. Large bottom-up driven bio-geographical shifts have been recorded in the Iceland basin, and changes in the strength and extent of the subpolar gyre have been associated with changes in four trophic levels – phytoplankton, zooplankton, blue whiting and pilot whales (*Globicephala melas*) [86]. The ecosystems of the Barents and Norwegian Seas are controlled by top-down and bottom-up forces: climate variability influences fish distribution, abundance, production, and growth rates. In addition, fish abundance is also controlled by complex interspecific interactions among fish species, particularly cod, capelin and herring, and their zooplanktonic prey, and predation by marine mammals [87]. The Barents Sea capelin stock underwent three collapses in the last 3 decades. The effects of the collapses propagated up and down the food web; zooplankton abundance increased due to release from predation pressure, and predators (cod, harp seals and seabirds) suffered negative consequences due to nutritional stress [88].

In this paper we have described the bottom-up forcers that regulate capelin in the Newfoundland-Labrador Shelf ecosystem. Although not a full account of energy flow regulation in the system, having advanced our understanding of the mechanisms that regulate the key forage species is a step in the right direction.

### Concluding Remarks

We have provided evidence for bottom-up control of energy flow in a wasp-waist marine ecosystem, the Newfoundland and Labrador Shelf, driven by physical factors through at least three trophic levels: from primary producers to zooplankton to forage species. Given the central role of capelin as a keystone species, it is expected that the bottom-up control would reverberate through the food web to the major predatory species. The implications of this finding are far-reaching in terms of achieving sustainable fisheries at the ecosystem level, i.e. not just for better defining capelin management practices, but also for delineating strategies that would promote recovery for higher trophic level species [89]. Traditional fisheries management has focused on target species, assuming that the main driving forces of exploited stocks are the fisheries themselves. Rarely are changes in productivity state and interactions among ecosystem components considered. Our results indicate that incorporating the impacts of environmental forcing on ecosystem productivity is a fundamental basis on which to develop Ecosystem-Based Management approaches [90]. Our findings are also relevant under the light of climate change predictions of general warming in the area [91]. It is unclear how the dynamics of seasonal sea ice will be affected and in turn how this will affect the system’s primary productivity. Climate change may elicit non-linear responses affecting patterns of synchrony among system components that can fundamentally change the energy flow and structure of the Northwest Atlantic food web.
